# Fluid flow-induced bending of the primary cilium triggers a distinct intraciliary calcium flux in mesenchymal stem cells

**DOI:** 10.1186/2046-2530-4-S1-P22

**Published:** 2015-07-13

**Authors:** M Corrigan, K Lee, M Labour, C Jacobs, D Hoey

**Affiliations:** 1 Centre for Applied Biomedical Engineering Research, University of Limerick, Limerick, Ireland; 2 Dept. Mechanical, Aeronautical and Biomedical Engineering, University of Limerick, Limerick, Ireland; 3 Materials and Surface Science Institute, University of Limerick, Limerick, Ireland; 4 Department of Biomedical Engineering, Columbia University, New York, NY, USA; 5 Trinity Centre for Bioengineering, Trinity College, Dublin, Ireland

## Objective

Primary cilia are proposed to form a regulatory microdomain in mesenchymal stem cells (MSCs) where they have also been shown to be necessary for loading-induced osteogenic lineage commitment. The specific mechanisms mediating this are unclear. This study aims to characterise subcellular ciliary calcium signalling in response to mechanical load and identify the mediating channels in MSCs.

## Method

Genetically encoded calcium indicators were transfected into murine MSCs. To analyse calcium within the ciliary micro-domain, we linked a sensor to a cilium specific GTPase (Arl13b). Oscillatory fluid flow was applied to model physiological mechanical loading. High speed real-time imaging of the sensors' was acquired. Ionomycin was added to the medium as a positive control. Immunocytochemical analysis of calcium channel incidence and localisation in MSCs was also carried out. Calcium channel expression was abrogated by transfection with siRNA targeting TRPV4 or PC2.

## Results

Upon fluid flow stimulation a peak in calcium signal was first detected within the cilium (Tpeak= 42s; Fold increase = 1.5) before a secondary peak in the cytoplasm (Tpeak= 44.5s; Fold increase = 2.5). TPRV4 and PC2 localise to the primary cilium of MSCs. TRPV4 or PC2 deficiency eliminates the response to flow throughout cells.

## Conclusion

Our data indicates that under fluid flow, there is a discrete calcium response within the cilium. TRPV4 and PC2 appear to facilitate this response which is believed to trigger a cascade of downstream events. These sites provide potential therapeutic targets to influence MSC differentiation.

